# Gynecological and Obstetrical Society of Baltimore

**Published:** 1893-04

**Authors:** William S. Gardner

**Affiliations:** Secretary; 613 Park Ave.


### Society Reports.

### GYNECOLOGICAL AND OBSTETRICAL SOCIETY OF BALTIMORE.

### DECEMBER MEETING.

The President, DR. B. B. BROWNE, in the chair.

DR. HUNTER ROBB read an abstract of an article by Prof. Gusserow on "Ascites in Connection with Gynecology."

In the Archiv fur Gynecologie for 1892, Prof. A. Gusserow, of Berlin, has an interesting article on "Ascites in Connection with Gynecology," of which the following is an abstract:

A high grade of ascites has often been observed occurring in connection with affections of the genital apparatus or of the peritoneum, which seem to occur by preference in women. In these cases at first even the skilled diagnostician cannot say anything more than that he has a general ascites (non-encapulated.) There is a lack of the symptoms which occur in ordinary ascites, there is no œdema in other parts, for instance, of the legs, the abdominal wall, or of the outer genitals. A patient often comes to us like a skeleton with the exception of a very prominent abdomen, which makes us think at once of an abdominal tumor in the modern sense of the word, i. e., a new growth. A special characteristic of this kind of case is the absence of all the ordinary factors, one or more of which are so often found to have given rise to ascites. So then in the first place, a careful examination must be made for disease of (1), the circulatory apparatus, (2), the liver, (3), the kidneys; and only those cases of ascites in which such etiological factors can be positively excluded, come properly speaking, into the domain of gynecology; and it is only these and none others that Gusserow is discussing. Most gynecologists are now agreed upon the best method of handling such cases. Unfortunately the ordinary practitioner is too apt to follow the older method, a circumstance which sometimes proves very unfortunate for the patient. He still clings to the idea that an attempt should be made to ascertain the cause of the ascites by means of a puncture, or what is worse, he is apt to make the treatment consist in further punctures and to continue these till the death of the patient. Puncture is in my opinion in every way inadvisable. It is true that we, in common with other gynecologists, for many years taught that puncture was always necessary for the diagnosis for an abdominal tumor. This idea they have now given up, and we consider it quite as absurd to make a puncture for diagnosis in the cases of general or "free" ascites. This new doctrine I have taught for years and the same holds good for tapping to take away the greater part of the fluid. It used to be the custom to make a puncture with a Pravaz "syringe," and draw off a little fluid, have it examined chemically and microscopically, in order to make a diagnosis of the kind of ascites and of its probable origin. Although much work has been done on the subject, there are many cases, and especially nearly all of these cases of "general ascites," which we are discussing now, where such an examination will give us no information at all. Better than this is tapping for the removal of the greater part of the fluid, since we thus get a better chance for palpation of the abdominal and pelvic organs, and may possibly be able to detect the cause which was concealed by the amount of the fluid. This "chance" of making a diagnosis frequently led to the adoption of this treatment, which as we said, was often not the best for the patient. The reasons against this method are: (1), the uncertainty of being able to make a diagnosis, even when the fluid is drawn off; (2), the faint chance, even with the best asepsis at our command, of setting up a septic process. (This latter danger has now, it is true, been reduced to a minimum, but we have nevertheless, seen cases of erysipelas and of septic peritonitis from tapping); (3), the liability of injuring vessels and of consequent internal bleeding; (4), the impossibility of drawing off all the fluid by tapping, and the almost certain return of the fluid which, perhaps, will necessitate tapping again and again.

I have given up both the puncture and tapping and prefer to make an incision about six cm., long, then empty the abdomen of the fluid; and inserting the finger, find out what is the local cause. One is then at once able to decide for or against an immediate or a future operation. If a radical operation is not to be done, we have at any rate drawn off all the fluid. The cases are divided into groups. To the first group belong cases of "general" ascites as a consequence of so called "tuberculous" peritonitis. This form appears mostly in young people. No lesion in the heart, kidneys, or liver is demonstrable and no signs of tuberculosis are anywhere found. On laparotomy one finds numerous nodules of a gray, reddish color both on the visceral and parietal surface of the peritoneum. Some of these cases, as it seems to us, are not cases of tuberculous peritonitis in the modern acceptation of the term. We would prefer to call them cases of "peritonitis nodosa." The first case given was observed by me twenty years ago, before the tubercle bacillus was discovered. The patient was quite young, twenty years of age; no signs of phthisis. There was a high grade of ascites. Patient had been tapped several times. Laparotomy performed, the fluid evacuted and the before mentioned nodules were found. Seeing the nodules I made a diagnosis of tuberculosis and gave a bad prognosis; and the patient—got well. In the second case laparotomy was performed, the fluid was evacuated and one of the nodules cut out. The central portion of the nodule was caseous, but no giant cells were found. (Tubercle bacilli had not been discovered and were not looked for). The patient recovered. The third case which was operated upon in 1892 was somewhat similar. The microscopical examination showed small celled proliferation with a rich blood supply. No giant cells, no tubercle bacilli. In any of these three cases tapping would have been of no avail, for it would not have been possible to make a diagnosis by palpation except by exclusion after the fluid was drawn off, and the diagnosis could only be established by opening the abdomen and cutting out one of the nodules for examination.

The second group consists of cases where the ascites was due to papilloma of the ovaries.

Papilloma of the ovary, or superficial papilloma of the ovary, consists of an abundant growth of connective tissue villi, which comes from the surface of the ovary, while the ovarian stroma itself is either found to be thickened or is nearly normal. These cases are not always distinguished from those rare cases in which a papilloma has burst, and a part of it has grown free in the abdominal cavity. The characteristics of superficial papilloma of the ovary are: (1), both ovaries are generally involved; (2), they cause a high grade of ascites, which is liable to return again after tapping; (3), they are generally too small to be palpated, even after tapping. The first observation of this kind was published by myself and Eberth in Virchow's Archiv, No. 43, 1868. Patient thirty-four years of age, had a high grade of ascites for a year and a quarter. Had been tapped several times (in Billroth's clinic among others). No reason for ascites discovered. Umbilical hernia developed and burst, and the patient increased the opening herself and let off the fluid. Finally the hernia became very large. A convolution of intestine had come out through the hernia, and when the patient was seen, most of the small intestine lay outside the abdomen and showed signs of discoloration. Operation for hernia. Rupture of gangrenous portion of intestine. Death. Postmortem showed: Papilloma of the ovary. (He also adds other cases). These cases of rare disease of the ovary ought to convince us that where we have ascites from some unknown cause in the abdomen, we ought not to limit ourselves to puncture, in fact we ought not to puncture at all. In none of them was it possible to diagnosticate the nature of the cause till the abdomen had been opened. In one of them where puncture had been made before, and bloody serous fluid evacuated, we might have been led to think of carcinoma of the peritoneum and been unwilling to operate. This admixture of blood, as a matter of fact, was a result of the puncture. In two of the cases death unfortunately followed the operation, but this must be attributed to the exhaustion of the patient by the frequent tappings.

In another case death was caused by septic peritonitis. Otherwise we feel sure that the patient would have been cured, since we have no instance of recurrent papilloma of the ovary where it has once been thoroughly excised.

To the third group belong those far more common cases of ascites due to carcinoma of the ovaries and the peritoneum. Here it might be asked, "is not incision unnecessary?" Here we can feel even a nodular tumor after puncture. Is it not sufficient to puncture in order to make the diagnosis." However, again we should employ incision. First, because we can never be otherwise sure that the growth is cancerous. Secondly, because only by this means can we decide whether (if there is carcinoma) the ovary or uterus ought to be removed, as it is the rule to extirpate cancerous ovaries unless the peritoneum is involved, and every carcinoma must be removed, if it is in healthy tissue. If there is carcinoma of the ovaries, then by incision we can tell whether or not the peritoneum is affected, and that can only be discovered by laparotomy. It will be objected that in malignant disease laparotomy has sometimes hastened death. This by no means always occurs, and against it we can put first, the certainty of diagnosis; secondly, if the tumor is benign a timely operation and recovery, and to these we may add, that where the tumor is malignant, a laparotomy sometimes hinders its progress, and even without further operation, life is prolonged.

These cases fall naturally into three subdivisions, (1), those in which the malignant growth could be removed (with ovaries). It must be remembered that we are not talking now of operations for malignant growths in the abdomen in general, but only of those in which general ascites was the characteristic symptom. Out of three cases two recovered completely, the third died later of multiple sarcomata. In the second subdivision come those cases in which the malignant growths could not be entirely removed. The first case has the following history: M. G., aged 20, admitted August 18, 1891. Primipara. Three months before entrance she had a great deal of pain in the abdomen, which obliged her to stay in bed. Was in bed four weeks. Before entrance she noticed a swelling, with no pain, but shortness of breath. The abdomen measured 110 cm. General ascites. No tumor felt by palpation or vaginal examination. Laparotomy, August 18, four to six litres of ascitic fluid removed. Tumor size of first on right side of uterus in layers of broad ligament. Mass adherent. Removed with difficulty because the tumor was of a friable, medullary material. A great deal of hemorrhage followed. Left ovary healthy. Diagnosis, spindle and round cell sarcoma. Patient recovered from the operation, but died of peritonitis without ascites, and of merasmus after seven months. Autopsy, general sarcoma of peritoneum, omentum, retro-peritoneal lymph glands, retro-sternal glands.

The next case one of carcinoma not connected with the genital apparatus, but adherent to the intestine. Removed. Patient left hospital completely well. She was lost sight of. Of the five cases in this catagory, in all which a portion of the growth was left in the abdomen, three died, not in consequence of the operation, but on account of the rapid development of the malignant growths. Two got well (one a woman of 75). What became of these cases ultimately is not known, any way their lives were prolonged by laparotomy.

To the last subdivision belong those cases of ascites where no attempt was made to remove the tumor, but where the abdominal section was made for the sole purpose of evacuating the fluid ; of five cases two died and three got better for the time being.

These cases show that for drawing off the fluid laparotomy is often better than puncture. The fluid can be so much better removed in this way, a diagnosis can be made, and we know with absolute certainty whether an operation is indicated or not.

Lastly we must mention cases of general ascites caused by benign disease of the genital apparatus. The first case was a woman, aged 57, nine children, came into clinic August 4th, 1890. Menopause one year ago, since that time had remarked a swelling in the abdomen, which caused her no particular inconvenience. For the last three weeks rapid increase in swelling, causing a feeling of tension, pain in abdomen and back with pain on micturition and prolapsus viginae (Abdomen 103 cm., from ascites). No tumor felt in abdomen; nothing discovered in the other organs. Laparotomy August 6th, 1890. Color of fluid yellow, hard tumor fastened to left cornu of uterus, casiny separated from it, right kidney a little out of place. The uterus was attached to abdominal wound. Tumor proved to be fibroma ovarii sinistri. Recovery. Vagina replaced. Even operation did not show the reason for so much ascites.

By tapping of course, we could not have discovered the real nature of the tumor causing the disease. We might indeed have felt the tumor, but could not have told about its malignant or non-malignant character. In another case belonging to this category we could not have had any idea of the nature of the disease by tapping. By laparotomy we were able to see plain indications for removal of the tumor, and were consequently able to cure the patient.

Prof. Gusserow in this article expresses, we think, the views of most of those of us who have had much experience in abdominal surgery. With our present technique, even were the advantages to be gained by such a procedure for less than they really are, we need not hesitate to open the abdomen instead of making a puncture. When the general practitioner meets with a case of ascites where all implication of the circulatory apparatus, the liver and kidney have once for all been definitely excluded, it would certainly be well for him to call in the specialist before adopting the "puncture" method. Our own experience fully bears out the futility of attempting to arrive at a certain diagnosis in every case by examination (chemical and microscopical) of the fluid which has been aspirated; the only certain way is to use the hand or the eye, or if possible both. Again we have seen more than one case which has come to autopsy where the patient had died after numerous aspirations and where the condition of things had led us to believe that a timely operation might have at least much prolonged the patient's life, even if the disease could not have been thoroughly eradicated. In these cases it seemed that valuable opportunities had been lost, and since an abdominal section not only gives the patient the best chance for complete recovery, but when employed as a palliative measure is more efficient than frequent tappings, it is in almost every case the better method. With respect to the "Peritonitis Nodosa" of which Gusserow speaks, it does not seem clear to us that these were not instances of peritoneal tuberculosis, and if this were the case the success which attended the operations and which we have seen confirmed in our work would only go still further in proving his proposition.

Finally in these days when in both medicine and surgery we are all striving as much as possible to avoid working in the dark, and we wish to treat our patients for the disease they really have and not for a hundred and one others which they might possibly be suffering from, the advantages of an absolute diagnosis can hardly be over-rated.

DR. J. WHITRIDGE WILLIAMS: I think we should take the case of Dr. Ashby's as a text to teach us to examine carefully for pelvic growths in all cases of ascites where heart and liver troubles are excluded. If we find ascites associated with a pelvic tumor the rational treatment is laparotmy and removal of the growth. The operation is very little more dangerous than puncture and is more satisfactory. Ascites is often due to tubercular peritonitis, papillomatous growths on the surface of the ovary, and solid tumors of the ovaries and tubes, both malignant and benign.

DR. B. B. BROWNE: These cases recall a case which I saw about three years ago of large accumulation of ascitic fluid in which it was quite impossible to make out any pelvic tumor. Kidney and heart diseases were excluded and the conclusion was come to that the ascites must be due to some intra-abdominal growth. An abdominal section was made, and after the evacuation of several gallons of ascitic fluid a papilloma of the ovary was found and removed. I saw the patient about three months ago; she was perfectly well.

DR. THOS. OPIE: I had a case of dropsy associated with large uterine fibroids. I made an abdominal incision with the intention of removing the fibroids, but finding that impracticable, the abdominal cavity was thoroughly sponged out and the wound closed. The patient was very much improved.

In many cases the simple opening of the abdominal cavity seem to at least temporarily relieve the patient, but the field is so new that we cannot assert positively just what permanent good can be attained by these operations.

DR. WM. E. MOSELY: Dr. Ashby's case recalls one which I reported fully some time ago. The patient had ascites and I aspirated her twice. Both times the abdomen rapidly refilled with fluid. I then opened her abdomen and removed two papillomatous growths. Three years have now elapsed since the operation; there has been no return of the ascites although the growth has returned.

DR. B. B. BROWNE: The question which is brought out most permanently is the propriety and advisability or an exploratory incision in those cases of ascites in which (heart, liver and kidney diseases having been excluded) no intrapelvic tumor can be felt.

Of course where the tumor is large enough to be mapped out, the accumulation of ascitic fluid no longer obscures the diagnosis or interferes with any operative procedure which would be called for were no fluid present.

WILLIAM S. GARDNER,
613 Park Ave. Secretary.

